# Biophysical constraints on mRNA decay rates shape macroevolutionary divergence in steady-state abundances

**DOI:** 10.1101/2025.11.24.690267

**Published:** 2025-11-24

**Authors:** Catherine Felce, Alexander L. Cope, Joshua G. Schraiber, Madhumitha Krishnaswamy, Lior Pachter, Matt Pennell

**Affiliations:** 1 Division of Physics, Mathematics and Astronomy, California Institute of Technology, Pasadena, CA, USA; 2 Department of Biological Sciences, Vanderbilt University, Nashville, TN, USA; 3 Evolutionary Studies Initiative, Vanderbilt University, Nashville, TN, USA; 4 Department of Quantitative and Computational Biology, University of Southern California, Los Angeles, CA, USA; 5 Department of Computational Biology, Cornell University, Ithaca, NY, USA; 6 Division of Biology and Biological Engineering, and Department of Computing and Mathematical Sciences, California Institute of Technology, Pasadena, CA, USA

**Keywords:** Biophysical modeling, single-cell transcriptomics, phylogenetics, evolutionary theory

## Abstract

Evolutionary changes to gene expression are understood to be a major driver of phenotypic divergence between species. Researchers have investigated the drivers of this divergence by fitting evolutionary models to multi-species ‘omic’ datasets. It is now apparent that steady-state mRNA expression levels show patterns consistent with evolutionary constraints, likely as a consequence of stabilizing selection. However, as all previous work has used bulk RNA measurements, it has been impossible to determine which of the many cellular processes that contribute to steady-state abundances underlie the divergence between species. Here we develop a novel paradigm for addressing this open problem. Using multi-species single-cell expression data and biophysical models, we estimate mRNA transcriptional burst sizes, splicing rates and decay rates across multiple species. We then derive phylogenetic models that describe the divergence of these rates under alternative evolutionary scenarios and fit these to the comparative data. We find evidence for biophysical constraints on the rates of mRNA decay, such that macroevolutionary divergence in expression is primarily a consequence of variation in transcriptional bursting.

## Introduction

Gene expression divergence is understood to be a key determinant of phenotypic divergence between species^1–3^. Omics technologies enabled comparative analyses of gene expression across individuals and species, providing insights into the molecular and evolutionary mechanisms shaping gene expression evolution, with most studies focusing on mRNA expression evolution measured via RNA-seq. Numerous studies have investigated gene expression evolution across species, revealing both widespread stabilizing selection and lineage-specific adaptive shifts in mRNA expression levels^4–8^. Furthermore, this data has been used to identify complex evolutionary patterns such as organ-specific evolution^6,9^, conserved gene regulatory modules^10^, differential expression correlated with the emergence of complex phenotypes^11^, and gene-by-gene coevolution of gene expression^12^. Recently, Cope et al.^13^ developed a phylogenetic framework to explicitly model the coevolution of mRNA and protein levels, revealing the mutational and selective coupling between these two layers of gene expression, and finding that natural selection is generally stronger on mean protein expression levels.

Despite the progress made in identifying patterns of gene expression evolution and the processes that drive them, these studies have been limited by their use of bulk mRNA measurements. While mean expression levels are a useful measure for studying gene expression evolution, these obfuscate *how* expression levels evolve and which mechanisms of gene expression are most dynamic or most constrained by natural selection. Steady-state mRNA abundances are determined by a large array of cellular processes^14–19^, which can make different contributions to between-species expression divergence. For example, experimental work using a two-species yeast hybrid found that changes to mRNA degradation rates were often accompanied by opposite-effect changes in transcription rate^20^. This implies that the evolution of regulatory elements has a multifaceted effect on gene expression levels^21–23^ that is strongly dependent on interactions between these elements across the genome, suggesting coevolution of regulatory mechanisms^24–26^.

To access information about these important regulatory mechanisms from transcriptomic data, we need new approaches that explicitly consider biophysical parameters. Principled biophysical modeling is crucial for extracting biological information from RNA-seq data^27^. The emergence of single-cell RNA-seq technologies has allowed for the estimation of key biophysical parameters, such as transcriptional burst size and frequency^28–30^. Single-cell snapshot experiments can effectively provide two ‘time points’, via the analysis of nascent and mature transcripts^31–33^. Such models have been used to estimate the relationship of transcriptional bursting to cell-cycle stage^34^, for principled cell-type clustering^35^, and to disentangle biological and technical noise^36^. The quantification of biophysical parameters from single-cell data opens up new routes for investigating mRNA expression evolution at the levels of transcriptional and post-transcriptional dynamics. With the increased availability of cross-species single-cell datasets, comparative analyses of biophysical parameters could reveal new insights into the biophysics of gene expression evolution on macroevolutionary timescales.

**Figure 1:**
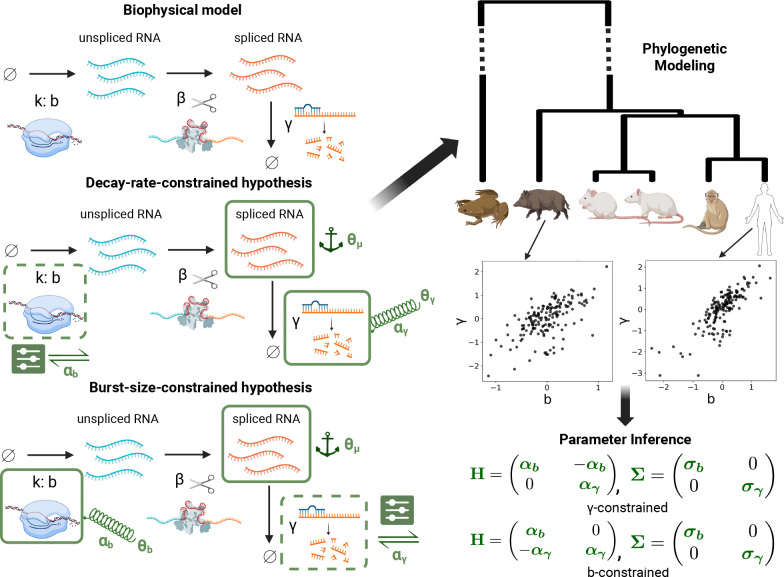
Our evolutionary model selection framework. The competing hypotheses impose different constraints on the evolution of the bursting size b and degradation rate γ. The model corresponding to each hypothesis is fit to the derived b and γ values for the species on the phylogenetic tree. Fitted selection matrices are obtained, and AICs suggest support for the decay-rate-constrained model.

In this work, we introduce a new paradigm for investigating the evolutionary interplay of different regulatory ‘levers’. Instead of bulk RNA measurements, we use single-cell data, fitting them with biophysically meaningful parameters. Specifically, we consider evolutionary hypotheses involving the coevolution of transcription and mRNA degradation across vertebrates (Figure 1). Motivated by experimental results,^20^, we posit a fitness landscape that gives rise to constraining selection on the mean level of spliced mRNA expression. However, when selection acts only on mean expression, the underlying biophysical processes that result in mean expression may evolve as if unconstrained, a process known as systems drift^37–40^. Thus, we investigate models in which one of the biophysical parameters is under constraining selection, whilst the other is free to adapt to maintain the optimal level of spliced mRNA, to test the hypothesis that selection acts both at the level of gene expression and at the level of the biophysical determinants of gene expression. Building upon the methods of Cope et al.^13^, we investigate the joint adaptation of transcriptional burst size and mRNA decay rate, finding support for the model with stabilizing selection on mRNA decay rates and mean spliced expression. This suggests that between-species differences in mRNA levels can be largely attributed to the flexible adaptation of transcriptional burst sizes.

## Results

For our basic biophysical model, we chose bursty transcription with the following dynamics:

(1) $$\emptyset \overset{k\;}{\to }B\times U\;U\overset{\beta \;}{\to }S\;S\overset{\gamma \;}{\to }\emptyset ,$$

where transcriptional bursts occur at a rate k, producing bursts of unspliced (U) transcripts with sizes distributed according to B, a geometric distribution with mean size b. The unspliced transcripts are converted to spliced mRNA (S) at a rate β, and the spliced transcripts then decay at a rate γ. The ∅ symbol indicates that the transcript before transcription and after decay does not appear in the model.

**Figure 2:**
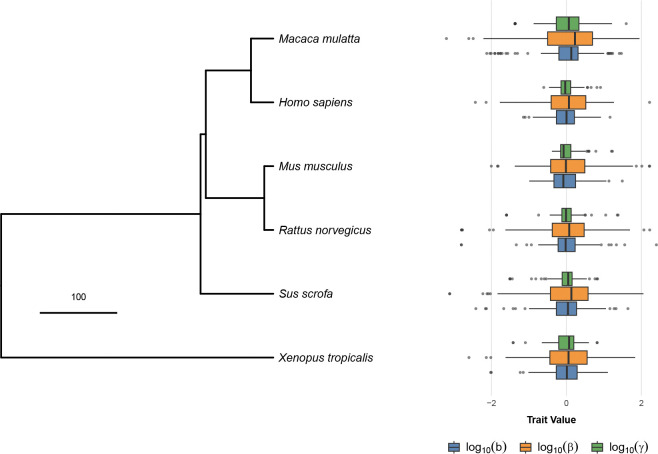
Values of the biophysical parameters across the phylogeny. The phylogeny is taken from TimeTree^43^ (scale bar in Myr). The boxplots at each tip show the mean-centered parameter distributions over genes for mean burst size, b, splicing rate, β, and RNA decay rate, γ, in the corresponding species. These trait values are the inputs into our phylogenetic model.

The biophysical parameters, b,β and γ, can be estimated from single-cell data using Monod^41^, which optimizes the likelihood of the observed counts under the bursty model. We used Monod to infer these biophysical model parameters for single-cell transcriptomics data from Jiao et al.^42^ across the spleens of individuals from six different species. The output from this procedure were per-gene, per-species values of the biophysical parameters (b,β,γ) across 167 orthologous genes (Figure 2).

We then developed a combined biophysical and phylogenetic model describing the evolution of log burst size (log(b)) and log mRNA decay rate (log(γ)) along a tree. We chose these two parameters because together they determine the log mean spliced mRNA level

(2) log(μ)=log(b)-log(γ),

which is independent of the splicing rate, β (note that γ is given in units of the burst initiation rate, k). Considering these two parameters allows us to test for a constrained version of quantitative systems drift^40^ (recall that the log burst size and log mRNA decay rate would be free to drift if selection only acted on log mean expression). In the Supplementary Information S2.1, we show that a two dimensional Ornstein-Uhlenbeck model captures the coevolution of burst size and mRNA decay rate due to selection on the mean spliced expression and an additional constraint on one of the two biophysical parameters. Thus, we can determine the biophysical mechanism through which gene expression evolution is mediated, and test which of the contributing biophysical processes is most constrained, alongside selection on mean expression.

We adopt a hierarchical model across genes, to exploit the large number of measured genes and compensate for the limited number of species in our dataset. In particular, while we assume that evolutionary rates are shared among genes, we allow the *optimal* biophysical parameters to vary between genes; in the Supplementary Information S5 we show that we can analytically integrate over a Gaussian prior on the optima. When sharing information across genes, due to lineage specific adaptation as well as biological and technical noise, some genes may not have any phylogenetic signal^44–46^. Thus, we assume that with probability $p_{wn}$ the biophysical parameters for a gene are taken from a white-noise distribution (see the outlier model in Chaix et al.^47^), and with probability $1-p_{wn}$ that they evolve corresponding to our coevolutionary model (see Methods for more details on the full model).

We used the logarithms of the biophysical parameters as continuous characters in our two hypothesized OU models (Figure 1; see Supplementary Information S2.1 for the derivation of these models from fitness landscapes). The first model, which we henceforth refer to as the decay-rate-constrained (γ-constrained) model, assumes that the primary form of selection is stabilizing selection on the mRNA decay rates. The burst size is then assumed to adjust in response, to achieve an optimal mean level of spliced mRNA expression. In this model, the selection matrix, H, takes the form:

(3) $$H=\left(\begin{array}{cc} \alpha_{b} & -\alpha_{b}\\ 0 & \alpha_{\gamma } \end{array}\right).$$

The second model, which we refer to as the burst-size-constrained (b-constrained) model, assumes that the fitness is most sensitive to the average value of the transcriptional burst size, b. The value of b therefore undergoes strong constraining selection, whilst the decay rate is assumed to adapt to maintain the optimal mean level of spliced mRNA expression. In this model, the selection matrix takes the form:

(4) $$H=\left(\begin{array}{cc} \alpha_{b} & 0\\ -\alpha_{\gamma } & \alpha_{\gamma } \end{array}\right).$$

We used simulations to assess the suitability of our model for inferring evolutionary parameters from biophysical parameters. The simulation results confirm that we can reliably distinguish between the two models described above (see Supplementary Information S2.3, Figures S1–S4).

### The data support a decay-rate-constrained model of transcriptional evolution

**Figure 3:**
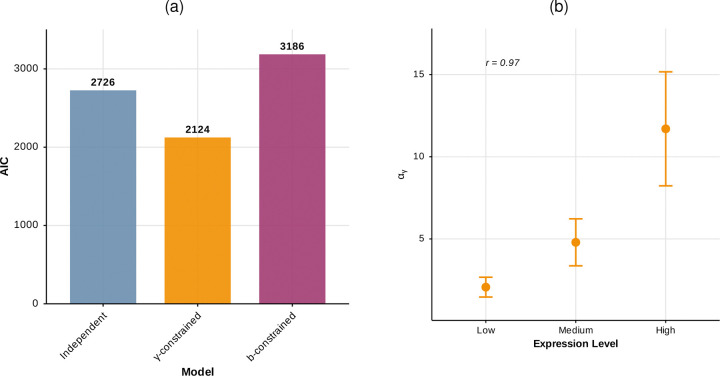
**(a):** AIC comparison across the tested phylogenetic models. A lower AIC value indicates a better model fit. **(b):** Selection coefficient for decay rate, $\alpha_{\gamma }$, in the decay-rate-constrained model, for gene sub-groups (n=56,55,56) binned by expression level.

After fitting both phylogenetic models to the average burst sizes, b, and decay rates, γ, extracted from the Jiao et al.^42^ data, we compared AIC values and found support for the decay-rate-constrained model (AIC=2124), over both the independent (AIC=2726) and burst-size-constrained (AIC=3186) models. The fit parameters are shown in Table 1, and the AIC values are shown in Figure 3a. The out-performance of the γ-constrained model over the independent model confirms the importance of modeling the *coevolution* of biophysical parameters.

There are numerous lines of evidence indicating that more highly-expressed genes generally experience stronger selection pressures, such as stronger purifying selection on amino acid substitutions^48,49^, stronger bias towards fast/accurate codons^50,51^ , and more conserved *cis* regulatory elements^52^. In previous work, Cope et al. found that high-expression genes exhibited stronger selection on mRNA levels compared to low-expression genes. We decide to verify this observation by using the AIC-preferred γ-constrained model to compare the driving selection rate, $\alpha_{\gamma }$, across genes with varying expression levels. To test whether selection on mRNA decay rates is stronger in more highly expressed genes, we binned the genes under investigation based on their median expression levels in human spleens^53^ and fit our phylogenetic mixture model separately to the genes in each bin, obtaining three sets of evolutionary parameters (Figure 3b). Additionally, we computed dN/dS values for the genes across the six species tree and found that, as expected, the genes with higher expression are subject to stronger purifying selection than those with lower expression (See Supplementary Information S2.5,Figure S5). These results suggest that our fitted selection rate correlates with mRNA expression level, consistent with other patterns observed in protein-coding sequence and gene regulatory evolution.

## Discussion

Our results reveal that the best model for mRNA expression evolution explicitly models the coevolution of mRNA burst size and decay rate. This accords with computational and experimental evidence that transcriptional evolution is a coordinated process across all regions of the genome^26^, and that burst size and decay rates evolve in a compensatory manner^20,54,55^. We further show that this coordinated model should involve constraining selection on the decay rate, with burst sizes then adapting to maintain the desired overall expression level. This is consistent with the pleiotropy of the mechanisms of decay-rate adaptation, which suggest that decay rates may be under stabilizing selection^56^ independently from transcriptional burst sizes. For example, there is a close connection between mRNA decay and translation^57–63^. This implies that protein-level constraints may also cause stabilizing selection on RNA decay rates. In addition, alternative 3’UTRs, another determinant of RNA stability, simultaneously affect membrane protein localization^64^, mRNA localization and translational efficiency^65^. This suggests that decay rates cannot adapt freely to achieve a certain level of expression, without also affecting other cellular processes.

In contrast, regulatory elements responsible for transcription are known to evolve rapidly^66^. For example, flexibly evolving promoter regions are thought to underlie a significant proportion of phenotypic diversity in humans^67^, and the frequent complete turnover of functional promoters has been observed in both humans and mice^68^. These promoter regions are directly related to transcriptional burst size^69,70^. Perhaps more so than promoters regions, comparative analysis reveal enhancer regions to experience rapid evolution^71,72^, with multiple studies indicating that enhancers play an important role in regulating the frequency of transcriptional bursting^69,73–75^. Chromatin state in regulatory regions can also be modified to influence transcriptional dynamics^76^, and has been shown to vary widely across human individuals^77^.Chromatin state therefore represents another free ‘tuning knob’ which can affect transcriptional burst size.

Overall, this work represents a new paradigm for probing the regulatory mechanisms underlying macroevolutionary mRNA expression divergence. For the first time, we combine principled biophysical modeling on single-cell data across species with phylogenetic comparative modeling. By selecting a multivariate OU model for biophysically meaningful parameters, we have investigated the selective coupling of these traits, giving insight into how transcription and decay rates coevolve. We have expanded on existing phylogenetic techniques, incorporating a multivariate OU model into a phylogenetic mixture model accounting for genes with no phylogenetic signal, as well as applying biophysical single-cell modeling in a coherent cross-species framework.

The approach outlined here can be extended to incorporate more complicated dynamics from both the biophysical and phylogenetic perspectives. As additional single-cell data modalities become available, with their corresponding joint biophysical models, these can be straightforwardly integrated into our method. For example, joint models for single-cell RNA with protein counts^78^ could be used to provide insight into the coevolution of translation and protein decay rates, along with the existing transcriptional rates. This would represent another avenue for corroborating the coevolutionary model proposed by Cope et al.^13^. In addition, including an integrated biophysical model for RNA and chromatin accessibility measurements^79^ could allow us to investigate the contribution of evolving on/off rates to gene expression evolution in a full telegraph model. The power of these approaches will increase as higher quality single-cell data, including more combined modalities across a greater number of species, become available.

On the phylogenetic side, our framework could be adapted to include more complicated evolutionary models, for example including mutational coupling between biophysical parameters, or expanding the dimensionality of the OU model to include coevolution between additional traits. With the flexibility to adapt and extend the two halves of our combined approach, researchers will be able to further dissect the contributions of different regulatory processes to the evolution of gene expression. This will provide new insights into how gene regulation has been shaped over the tree of life.

## Methods

### Data processing and biophysical modeling

For this study we use single-cell RNA-seq data from Jiao et al.^42^, extracted from the spleen of seven different species. We processed the data using kallisto^81–84^ to obtain spliced and unspliced count matrices. After clustering the data from each species by cell-type, we excluded the fish sample from further analysis because of an indistinct and low-count T-cell cluster, leaving six remaining species, which were filtered for T-cells. We searched for genes which had orthologs in all six species using Ensembl BioMart^85^.

We then fit transcriptional rates for these genes in each species separately using Monod^41^, using the bursty transcription model with Poisson technical noise. Monod is an inference framework which fits per-gene biophysical parameters for a selection of transcriptional models. The dynamics of the model are encapsulated in a chemical master equation, which can then be numerically solved to give steady-state distributions for spliced and unspliced RNA counts, including the impact of technical noise. The likelihood of the observed spliced/unspliced count matrices can then be maximized over biophysical parameters. In practice, this process is repeated over a grid of technical parameters, and the combined values which maximize the likelihood of the data are outputted.

The output of this procedure is a per-gene burst size, b, splicing rate β, and decay rate, γ, with the rates given in units of the transcription initiation rate, k, all in log space. We then subtracted the mean of each parameter across genes from each species. After fitting with Monod, which filters some genes, and removing genes without a fitted ortholog in all six species, we were left with 167 genes. The mean-centered log values of b,β and γ for each of these genes were used as the traits for the following analysis.

### Phylogenetic modeling and parameter inference

We consider the two-dimensional Ornstein-Uhlenbeck models for burst size, b, and decay rate, γ described in Results. Under these models, the logarithms of b and γ follow the following evolution equations:

(5) $$dX_{t}=-H(X_{t}-\overset{ˆ}{X})dt+\Sigma dW_{t},$$

where $W_{t}$ is a Wiener process. In our models, we set Σ as a diagonal matrix with entries $\sigma_{b}$ and $\sigma_{\gamma }$. $X_{t}$ represents the logarithms of the two biophysical rates:

(6) Xt=logbtlogγt

The forms for the selection matrices in each model,

(7) $$H=\left(\begin{array}{cc} \alpha_{b} & -\alpha_{b}\\ 0 & \alpha_{\gamma } \end{array}\right)\;\text{and}\;H=\left(\begin{array}{cc} \alpha_{b} & 0\\ -\alpha_{\gamma } & \alpha_{\gamma } \end{array}\right),$$

are derived from the following forms for the fitness function, w:

(8) $$w(b,\gamma )\propto exp\left(-\frac{{\left(\overset{ˆ}{\gamma }\;-\;\theta_{\gamma }\right)}^{2}}{2V_{\gamma }}-\frac{{\left((\overset{ˆ}{b}\;-\;\overset{ˆ}{\gamma })\;-\;\theta_{\mu }\right)}^{2}}{2V_{\mu }}\right),\;exp\left(-\frac{{\left(\overset{ˆ}{b}\;-\;\theta_{b}\right)}^{2}}{2V_{b}}-\frac{{\left((\overset{ˆ}{b}\;-\;\overset{ˆ}{\gamma })\;-\;\theta_{\mu }\right)}^{2}}{2V_{\mu }}\right),$$

where we use $\overset{ˆ}{b},\;\overset{ˆ}{\gamma }$ for the logarithms of b,γ, and for optimal log parameter values, $\theta_{b,\gamma }$ and an optimal log spliced mean expression level, $\theta_{\mu }$. Note that, since the optima and parameter values are in log space, $\overset{ˆ}{b}-\overset{ˆ}{\gamma }$ represents the ratio of burst size to mRNA decay rate, which is proportional to the mean spliced expression level. See the supplementary information, Section S2, for the full derivation of these models, which follows Cope et al.^13^.

For parameter inference, we take the values of logb and logγ for each species, along with the phylogenetic tree. We fit a mixture model, where each gene is generated from a white noise distribution with probability $p_{wn}$, and from the relevant phylogenetic model with probability $1-p_{wn}$. The evolutionary selection strengths, $\alpha_{b,\gamma }$, and stochastic rates, $\sigma_{1,2}$, are constrained to be equal across genes, whereas the optima $\theta_{\mu }$ and $\theta_{b,\gamma }$ are assumed to be drawn from normal distributions whose parameters are optimized.

We also fitted an independent OU model using MCMC for all of the biophysical parameters (b,β and γ), whose details and results are included in the supplementary information, section S3 (Figures S6–S8). In addition, we fit two three-parameter-H versions of the coevolution model, whose results are included in the supplementary information, Section S4, Figures S9–S12.

## Supplementary Material

Supplement 1

## Figures and Tables

**Table 1:** Phylogenetic model fitted parameters: selection rates, $\alpha_{b,\gamma }$, and mutations rates, $\sigma_{b,\gamma }$, on average transcriptional burst size, b, and mRNA decay rate, γ, along with the probability for a gene’s biophysical parameters to be drawn from a white-noise distribution, $p_{wn}$.

Model	$\alpha_{b}$	$\alpha_{\gamma }$	$\sigma_{b}$	$\sigma_{\gamma }$	$p_{wn}$

γ-constrained	31.5	2.33	0.923	1.06	0.149
b-constrained	0.022	3.56	3.01	0.169	0.900
Independent	1.36	1.59	0.651	0.831	0.329

## Data Availability

The Genotype-Tissue Expression (GTEx) Project was supported by the Common Fund of the Office of the Director of the National Institutes of Health, and by NCI, NHGRI, NHLBI, NIDA, NIMH, and NINDS. The average expression data used for the gene binning described in this manuscript were obtained from the GTEx Portal, V10 spleen tissue, on 10/15/2025. The transcriptomic data used in our analysis is taken from Jiao et al.^42^. The code and data for the phylogenetic model used to perform these analyses, and scripts to reproduce Figures 2, 3 and the supplementary figures are available at https://github.com/pachterlab/FCSKPP_2025/tree/main^80^.
