# Supplementary material for: Biophysical constraints on mRNA decay rates shape macroevolutionary divergence in steady-state abundances
Source: bioRxiv. 2025 Nov 24:2025.11.24.690267. Preprint. [Version 1] doi: 10.1101/2025.11.24.690267 (PMC12697285; doi:10.1101/2025.11.24.690267)
Supplement: Supplement 1 [file NIHPP2025.11.24.690267v1-supplement-1.pdf]

bioRxiv preprint doi: https://doi.org/10.1101/2025.11.24.690267; this version posted November 24, 2025. The copyright holder for this preprint (which was not certified by peer review) is the author/funder, who has granted bioRxiv a license to display the preprint in perpetuity. It is made available under aCC-BY 4.0 International license.

Supplemental Information for: Biophysical constraints on mRNA decay rates shape macroevolutionary divergence in steady-state abundances

Felce *et al.*

S1 Data processing

S1.1 Pre-processing

For this study we use single-cell RNA-seq data from Jiao et al.[1], extracted from the spleen of seven different species. We processed the data using kallisto[2] to obtain spliced and unspliced count matrices, and filtered out low UMI cells. An example kallisto call is given here:

```
kb count --verbose -i ./frog/index.idx -g ./frog/t2g_mm10.txt -x 10xv2
    -o ./frog/output -t 24 -m 8G -c1 ./frog/cdna_t2c.txt -c2 ./frog/intron_t2c.txt
    --workflow=nac --filter bustools --strand=unstranded --sum=cell
    ../SRR16490736_1.fastq ../SRR16490736_2.fastq,
```

with example output summary:


```
"n_targets": 76913,
"n_bootstraps": 0,
"n_processed": 289062079,
"n_pseudoaligned": 190915538,
"n_unique": 35185121,
"p_pseudoaligned": 66.0,
"p_unique": 12.2,
"kallisto_version": "0.50.1",
"index_version": 13,
"start_time": "Wed Jun 12 15:04:30 2024"
```


After clustering the data from each species by cell-type, we excluded the fish sample from further analysis because of an indistinct and low-count T-cell cluster, leaving six remaining species, which were filtered for T-cells.

S1.2 Fitting biophysical parameters

We searched for genes which had orthologs in all six species using Ensembl BioMart[3]. We then fit transcriptional rates for these genes in each species separately using Monod[4], using the bursty transcription model with Poisson technical noise. After fitting with Monod, which filters some genes, and removing genes without a fitted ortholog in all six species, we were left with 167 genes. An example of the Monod run is given below:

bioRxiv preprint doi: https://doi.org/10.1101/2025.11.24.690267; this version posted November 24, 2025. The copyright holder for this preprint (which was not certified by peer review) is the author/funder, who has granted bioRxiv a license to display the preprint in perpetuity. It is made available under aCC-BY 4.0 International license.

```
fitmodel = cme_toolbox.CMEModel('Bursty','Poisson')
filt_param = {'min_means':[0.01, 0.01], 'max_maxes':[350, 350], 'min_maxes':[1,3]}

lb = [-1.0, -1.8, -1.8 ]
ub = [4.2, 2.5, 3.5]
# samp_lb, samp_ub = [-8, -3],[-5, 0]
samp_lb, samp_ub = [-11, -6],[-5, 0]

grid = [6,7]

fitted_adata = inference.perform_inference(combined_adata, fitmodel, n_genes=5000,
    seed=5, phys_lb=lb, phys_ub=ub, gridsize=grid,
    samp_lb=samp_lb, samp_ub=samp_ub, filt_param=filt_param,
    gradient_param={'max_iterations':5,'init_pattern':'moments','num_restarts':1},
    dataset_string=dataset_string, viz=True,num_cores=32)
```

The output of this procedure is a per-gene burst size, b, splicing rate β, and decay rate, γ, with the rates given in units of the transcription initiation rate, k, all in log space. We then subtracted the mean of each parameter across genes from each species, and used the resulting values as the traits for the phylogenetic analysis.

S1.3 Phylogenetic tree

For the phylogenetic tree, we used the following tree, from TimeTree[5], in Newick format:

```
(Frog:351.68654000,(Pig:94.00000000,((Rat:11.64917000,Mouse:11.64917000)
'14':75.55083000,(Human:28.82000000,Macaque:28.82000000)'13':58.38000000)
'25':6.80000000)'37':257.68654000);
```

S2 Two-dimensional evolution model

S2.1 Phylogenetic model derivation from fitness landscape

Following Cope et al.[6], we consider a fitness function of the form:

$$w(b,\gamma) \propto \exp\left(-\frac{(\log\gamma - \theta_\gamma - \phi_\gamma \log b)^2}{2V_\gamma} - \frac{(\log b - \theta_b - \phi_b \log\gamma)^2}{2V_b}\right). \tag{1}$$

Since transcriptional rates are the mechanisms by which cells control the level of transcription, we assume that mutations can affect b and γ independently. This corresponds to setting $c = 0$ in the model from Cope et al.[6]. Then, using their expression for the evolution matrix, (their F), we have:

$$H = \begin{pmatrix} \alpha_b(1+\phi_\gamma^2\omega) & -\alpha_b(\phi_b+\phi_\gamma\omega) \\ -\alpha_\gamma\left(\frac{\phi_b}{\omega}+\phi_\gamma\right) & \alpha_\gamma\left(1+\frac{\phi_b^2}{\omega}\right) \end{pmatrix}, \tag{2}$$

We then consider two different models. In the first model, we assume that selection acts first on the decay rate, γ, and then on the mean spliced RNA value, μ_s, via adaptation of the burst size,

bioRxiv preprint doi: https://doi.org/10.1101/2025.11.24.690267; this version posted November 24, 2025. The copyright holder for this preprint (which was not certified by peer review) is the author/funder, who has granted bioRxiv a license to display the preprint in perpetuity. It is made available under aCC-BY 4.0 International license.

b. This is equivalent to setting $\phi_\gamma = 0$ and $\phi_b = 1$, recalling that, in log space, $\log \mu_s = \log b - \log \gamma$. Note that, since the unspliced mean, μ_u, is given by $\frac{b}{\beta}$, and the spliced mean is given by $\frac{\mu_u \beta}{\gamma}$, the splicing rate β does not influenced the mean spliced counts, μ_s (recall that rates are in fitted in units of the transcriptional initiation rate). In this model, we also relabel $V_b \to V_\mu$ and $\theta_b \to \theta_\mu$, to emphasize that the pressure on b to adjust is equivalent to selection pressure on the mean spliced RNA level. So we have, for the fist, γ-constrained model:

$$w(b, \gamma) \propto \exp\left(-\frac{(\log \gamma - \theta_\gamma)^2}{2V_\gamma} - \frac{(\log b - \log \gamma - \theta_\mu)^2}{2V_\mu}\right). \quad (3)$$

For the γ-constrained model, H becomes:

$$H = \begin{pmatrix} \alpha_b & -\alpha_b \\ -\frac{\alpha_\gamma}{\omega} & \alpha_\gamma \left(1 + \frac{1}{\omega}\right) \end{pmatrix}, \quad (4)$$

and $\omega = \frac{V_\mu}{V_\gamma}$. In this γ-constrained model, we further assume that $V_\gamma \ll V_\mu$, such that we can neglect terms $\mathcal{O}\left(\frac{1}{\omega}\right)$, giving:

$$H = \begin{pmatrix} \alpha_b & -\alpha_b \\ 0 & \alpha_\gamma \end{pmatrix}. \quad (5)$$

For our second model, we assume that b is tightly constrained, and γ is more free to vary and adjust to the optimum mean RNA level. This corresponds to setting $\phi_b = 0$ and $\phi_\gamma = 1$. The fitness function then becomes:

$$w(b, \gamma) \propto \exp\left(-\frac{(\log b - \log \gamma - \theta_\mu)^2}{2V_\mu} - \frac{(\log b - \theta_b)^2}{2V_b}\right), \quad (6)$$

where we have relabeled $V_\gamma \to V_\mu$ and $\theta_\gamma \to -\theta_\mu$ (note the change of sign in the first term has no effect due to the squaring). This gives, as above:

$$H = \begin{pmatrix} \alpha_b(1+\omega) & -\alpha_b \omega \\ -\alpha_\gamma & \alpha_\gamma \end{pmatrix}, \quad (7)$$

For this, b-constrained, model, $\omega \equiv \frac{V_b}{V_\mu}$, and we assume $V_b \ll V_\mu$, giving:

$$H = \begin{pmatrix} \alpha_b & 0 \\ -\alpha_\gamma & \alpha_\gamma \end{pmatrix}. \quad (8)$$

We compare these models with a fully independent model (diagonal H), as well as more generic models. For all models, we fit a diagonal matrix for the stochastic term:

$$\Sigma = \begin{pmatrix} \sigma_b & 0 \\ 0 & \sigma_\gamma \end{pmatrix}, \quad (9)$$

which depends on the relative mutation rates of b and γ.

Recall Cope et al. also have:

bioRxiv preprint doi: https://doi.org/10.1101/2025.11.24.690267; this version posted November 24, 2025. The copyright holder for this preprint (which was not certified by peer review) is the author/funder, who has granted bioRxiv a license to display the preprint in perpetuity. It is made available under aCC-BY 4.0 International license.

$$\hat{\boldsymbol{X}} = \begin{pmatrix} \frac{\theta_b + \phi_b \theta_\gamma}{1 - \phi_b \phi_\gamma} \\ \frac{\theta_\gamma + \phi_\gamma \theta_b}{1 - \phi_b \phi_\gamma} \end{pmatrix}, \tag{10}$$

where, again, we have switched to our notation. Since either ϕ_b or ϕ_γ is zero in each of our models, the denominator is always equal to one. In particular, we have:

$$\hat{\boldsymbol{X}} = \begin{pmatrix} \theta_\mu + \theta_\gamma \\ \theta_\gamma \end{pmatrix}, \qquad \begin{pmatrix} \theta_b \\ -\theta_\mu + \theta_b \end{pmatrix}, \tag{11}$$

for the first and second models respectively. We then assume that the logarithms of b and γ evolve along the tree according to

$$d\boldsymbol{X}_t = -H(\boldsymbol{X}_t - \hat{\boldsymbol{X}}) + \Sigma d\boldsymbol{W}_t, \tag{12}$$

as in the independent case, where now

$$d\boldsymbol{X}_t = \begin{pmatrix} \log b \\ \log \gamma \end{pmatrix}, \tag{13}$$

and H, $\hat{\boldsymbol{X}}$ and Σ are as specified above. Note that the selection matrix H is the only structural difference between the two models.

S2.2 Evolutionary parameter inference

We fit a mixture model where a fraction p_{wn} of genes are assumed to be drawn from a white-noise distribution. The rest of the genes are assumed to be generated from the relevant phylogenetic model, described in S2.1.

The evolutionary parameters in H and Σ are assumed constant across genes, but the optimal values $\theta_\mu, \theta_{b,\gamma}$ are allowed to vary per gene, and are assumed to be drawn from a normal distribution centered at $\bar{\theta}^P_\mu$ and $\bar{\theta}^P_{b,\gamma}$ respectively, where the P denotes the population of optima over genes. The standard deviations of these populations of optima are also fit as part of the likelihood maximization ($\tau^P_{b,\gamma}, \tau^P_\mu$). We then analytically integrate over the possible values of θ_μ and $\theta_{b,\gamma}$.

The white-noise distribution is a two-dimensional Gaussian with mean $\hat{\boldsymbol{X}}$, with the values of $\theta_\mu, \theta_{b,\gamma}$ assumed to be drawn from the same distributions as for the phylogenetic model, and diagonal covariance matrix with entries $\sigma^{b,\gamma}_{wn}$. Since we analytically integrate over possible values of $\theta_{b,\gamma}$, this amounts to a new two-dimensional Gaussian distribution with means $\bar{\theta}^P_\mu$ and $\bar{\theta}^P_{b,\gamma}$, with covariance matrix given by Cov($\hat{\boldsymbol{X}}$) (see section S5).

Overall, this gives an 11-parameter model, with 4 evolutionary parameters, 3 white-noise parameters, 4 optima distribution parameters and the mixture probability p_{wn}. The likelihood of the data under the full model is optimized using `optimx`[7].

S2.3 Simulation results: Two-dimensional model

For the model comparison, we simulate 100 datasets under each of hypotheses 1 and 2 (decay rate and burst size-driven), using PCMBase[8]. We draw random sets of true parameters for each

bioRxiv preprint doi: https://doi.org/10.1101/2025.11.24.690267; this version posted November 24, 2025. The copyright holder for this preprint (which was not certified by peer review) is the author/funder, who has granted bioRxiv a license to display the preprint in perpetuity. It is made available under aCC-BY 4.0 International license.

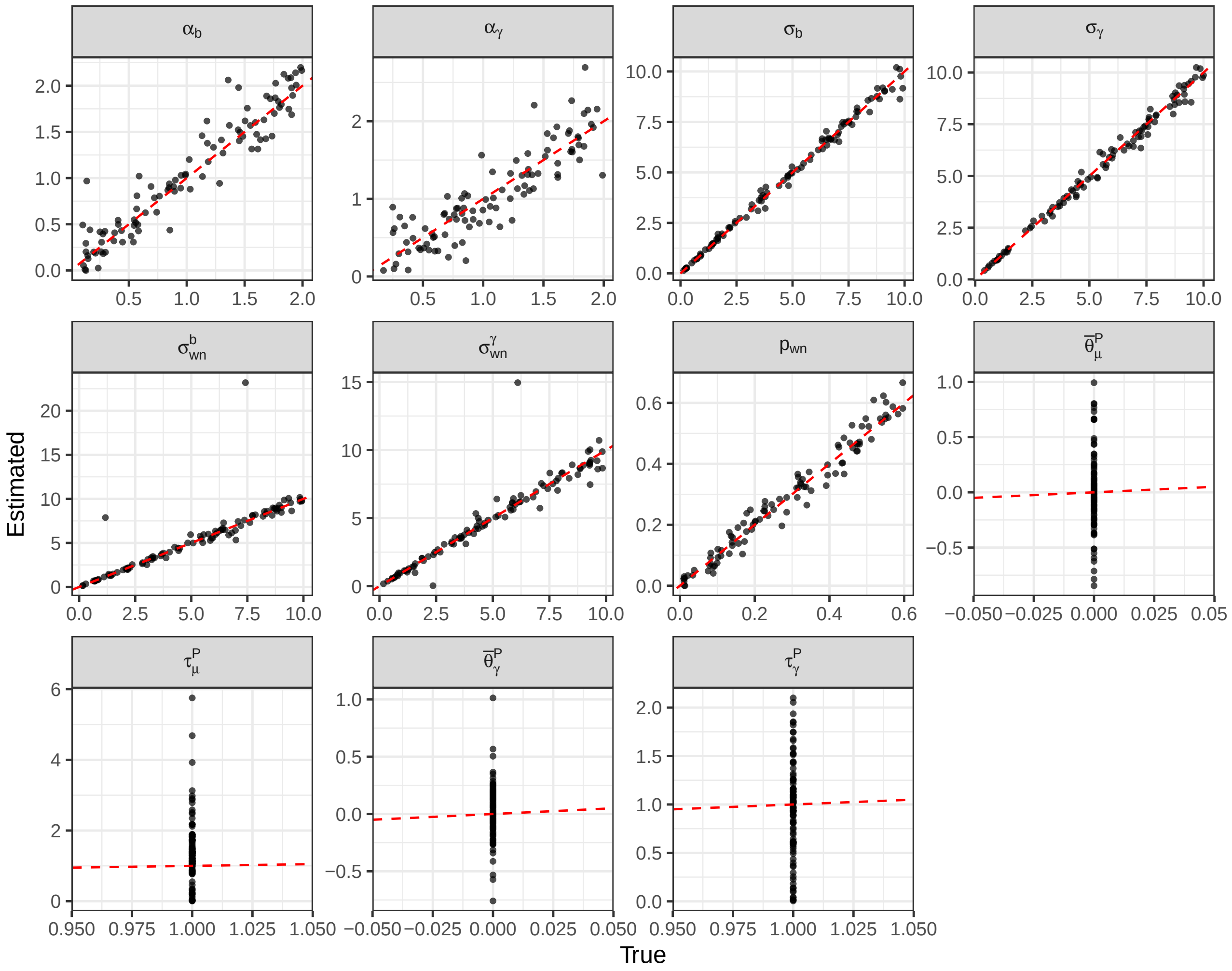

Figure S1: Fitted vs true parameters for the decay-rate-constrained model.

simulation uniformly between the bounds. We then fit the simulated datasets using the procedure described above. The results for the decay-rate-constrained model are shown in Figure S1, and the results for the burst-size-constrained model are shown in Figure S2.

We also performed a model comparison by simulating data under both models, and fitting both datasets under each model. We show the distribution of AIC differences in favor of the correct model in Figure S3. We show the corresponding accuracy of differentiating between models using the AIC value in Figure S4. This is given by the fraction of model fits with AIC differences above each cutoff which would be attributed to the correct model.

S2.4 Data fit details

We include the fitted parameter values for the two-parameter H, two-dimensional OU model in Table 1. Note that the burst-size-constrained model hits the upper bound for p_{wn}, the probability for each gene to be pure noise in our mixture model.

S2.5 dN/dS calculations

To look at the signatures of selection for the 167 genes in our dataset across the six species phylogeny, we computed dN/dS values. We adopted a standard bioinformatic pipeline, using protein

bioRxiv preprint doi: https://doi.org/10.1101/2025.11.24.690267; this version posted November 24, 2025. The copyright holder for this preprint (which was not certified by peer review) is the author/funder, who has granted bioRxiv a license to display the preprint in perpetuity. It is made available under aCC-BY 4.0 International license.

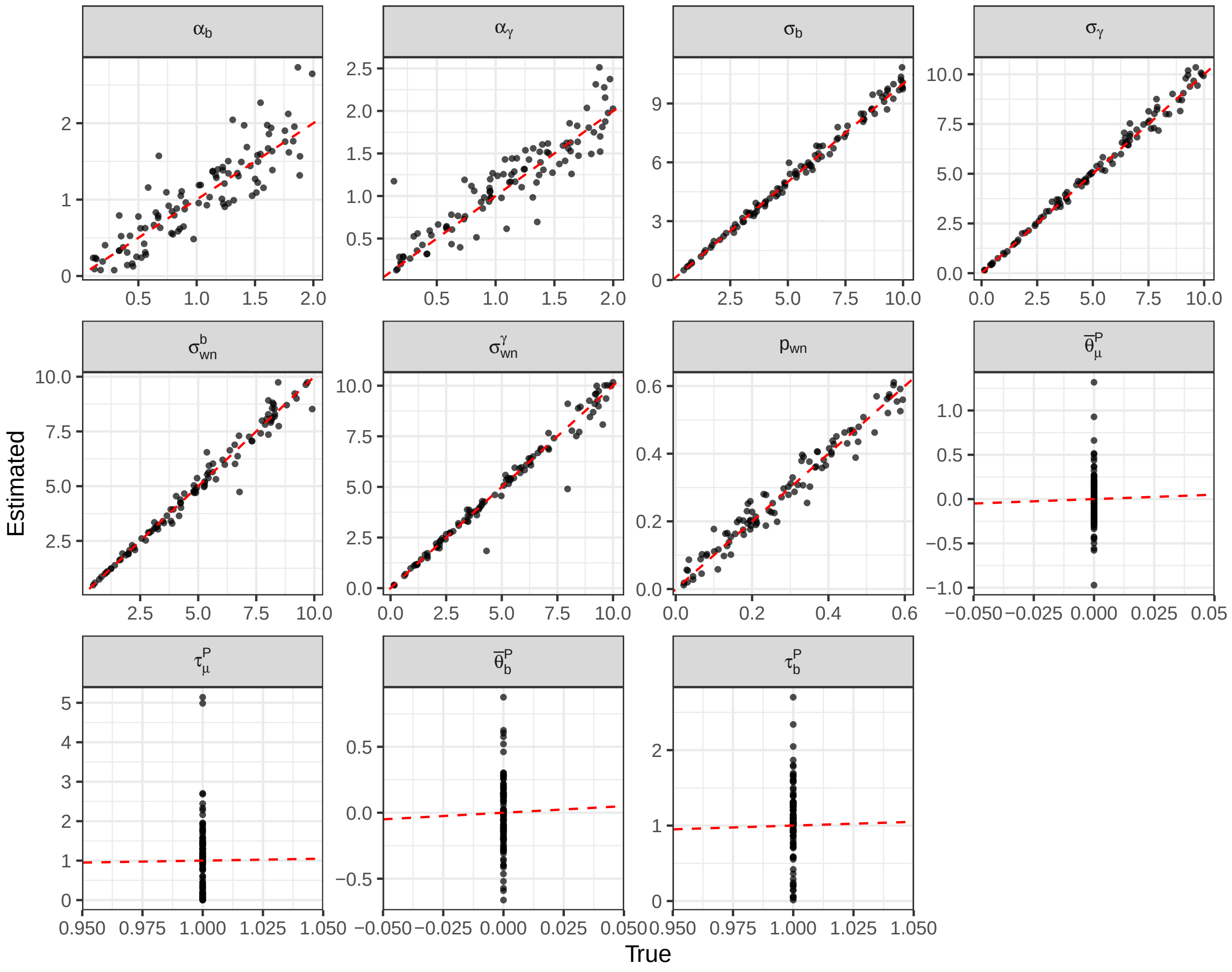

Figure S2: Fitted vs true parameters for the burst-size-constrained model.

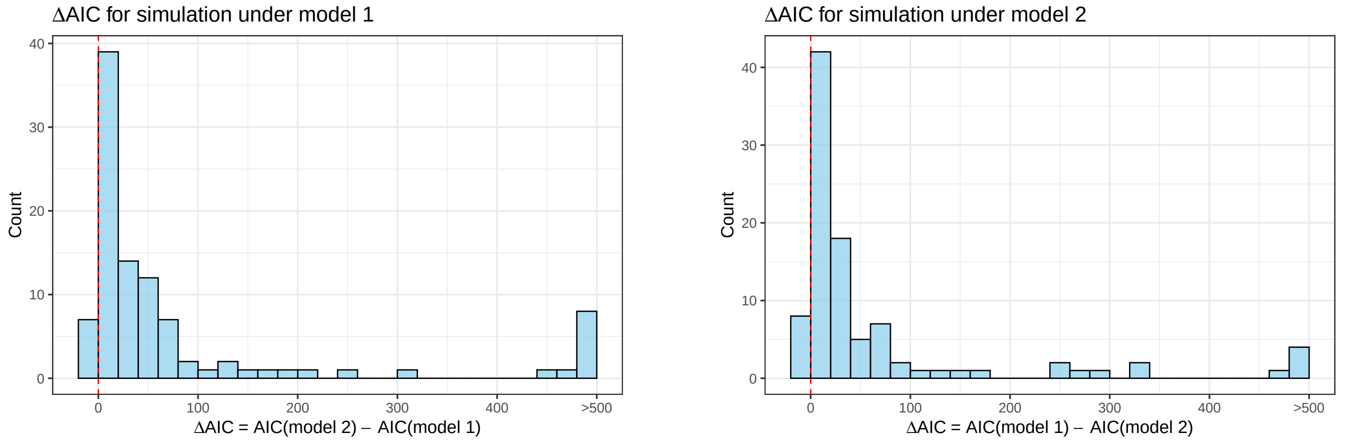

Figure S3: Distributions of AIC differences in favor of the correct model, for datasets simulated under the decay-rate-constrained model **(left)** and the burst-size-constrained model **(right)**, fitted under both models.

and cDNA sequences from Ensembl[9], protein alignment using MAFFT[10], codon based alignment with Pal2Nal[11] and dN/dS calculations using CODEML from PAML[12,13].Out of 167 genes, we obtained values for 159 gene ortholog groups which we plotted based on the bins of gene

bioRxiv preprint doi: https://doi.org/10.1101/2025.11.24.690267; this version posted November 24, 2025. The copyright holder for this preprint (which was not certified by peer review) is the author/funder, who has granted bioRxiv a license to display the preprint in perpetuity. It is made available under aCC-BY 4.0 International license.

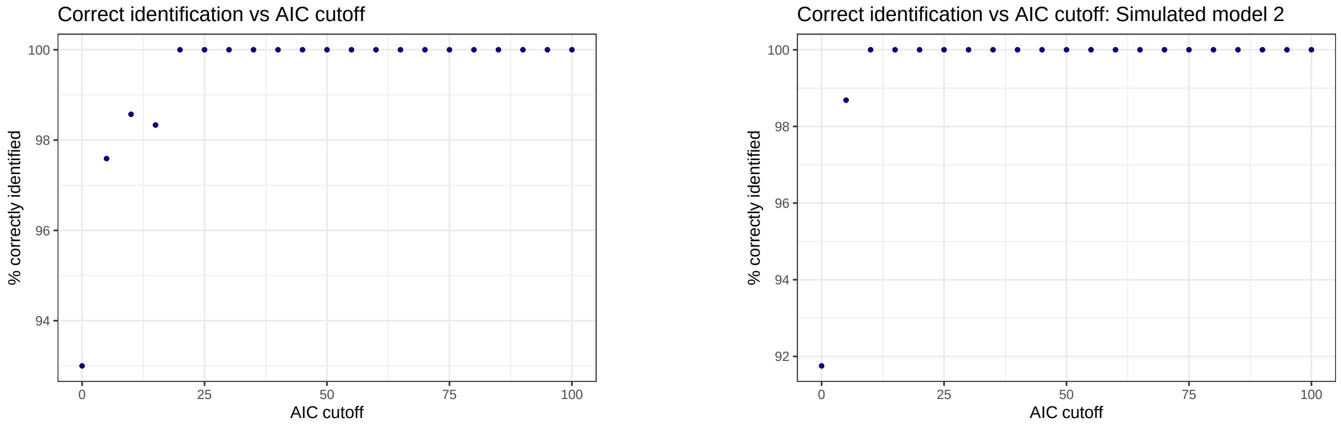

Figure S4: Accuracy of model identifications made at various AIC cutoffs, for datasets simulated under the decay-rate-constrained model **(left)** and the burst-size-constrained model **(right)**, fitted under both models. The accuracy is defined as the fraction of model fits with AIC differences above each cutoff which would be attributed to the correct model.

Table 1: Two-dimensional OU model fitted parameters

Model	α_b	α_γ	σ_b	σ_γ	p_{wn}	AIC
γ-constrained	31.5	2.33	0.923	1.06	0.149	2124
b-constrained	0.022	3.56	3.01	0.169	0.900	3186
Independent	1.36	1.59	0.651	0.831	0.329	2726

expression, shown in Figure S5.

S3 Independent evolution model

We also considered the simple case where, for each gene, the biophysical parameters, b, β and γ, evolve independently. We assume that each gene also evolves independently, but that all genes evolve according to the same evolutionary dynamics[14,6]. This amounts to a shared selection matrix, H, and mutation matrix, Σ, in the OU evolution equation:

$$d\boldsymbol{X}_t = -H(\boldsymbol{X}_t - \hat{\boldsymbol{X}}) + \Sigma d\boldsymbol{W}_t, \tag{14}$$

where $\boldsymbol{X}$ is given by the logarithms of the three biophysical rates,

$$\boldsymbol{X}_t = \begin{pmatrix} \log b_t \\ \log \beta_t \\ \log \gamma_t \end{pmatrix}. \tag{15}$$

The assumed independence of the biophysical rates is enforced by diagonality of H and Σ. We therefore define parameter-specific selections rates, α, and stochastic standard deviations, σ, via

bioRxiv preprint doi: https://doi.org/10.1101/2025.11.24.690267; this version posted November 24, 2025. The copyright holder for this preprint (which was not certified by peer review) is the author/funder, who has granted bioRxiv a license to display the preprint in perpetuity. It is made available under aCC-BY 4.0 International license.

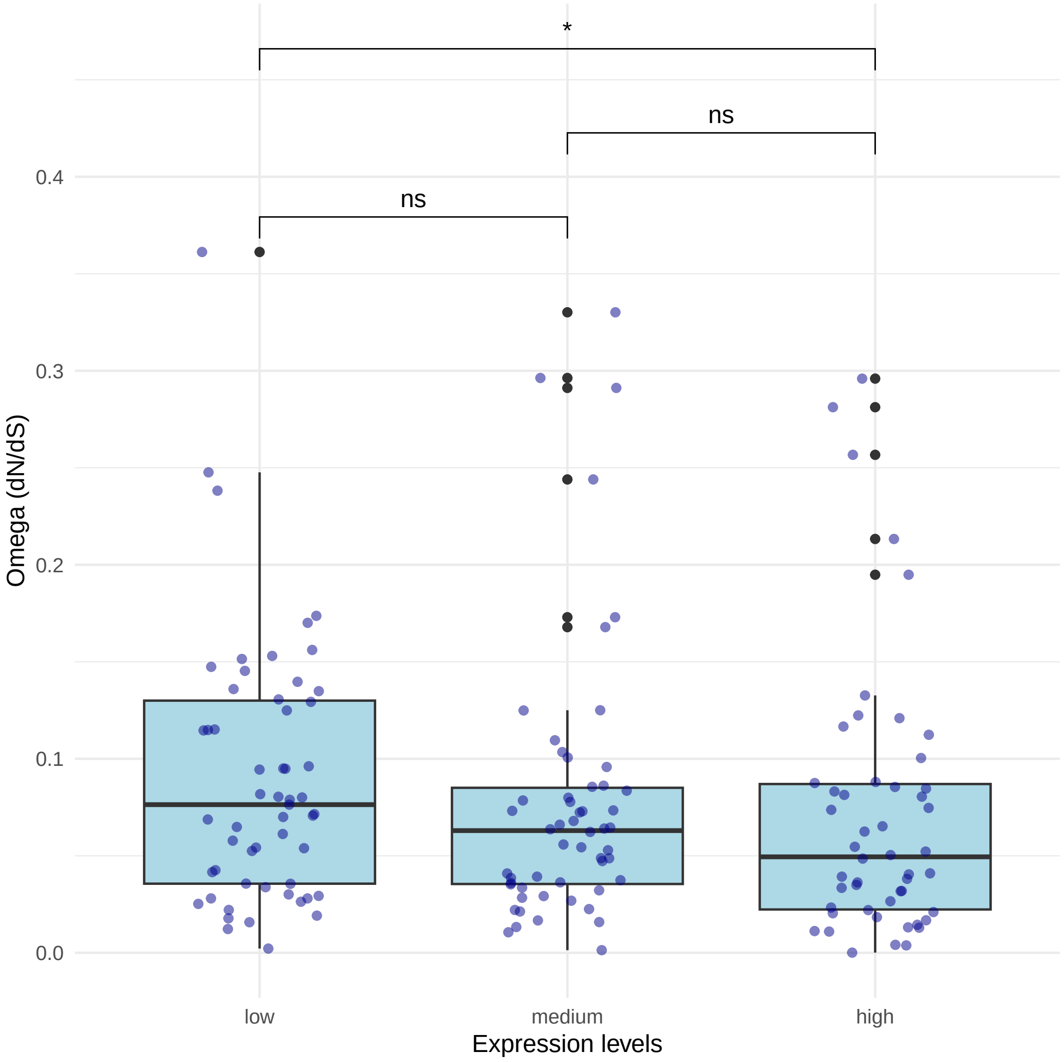

Figure S5: dN/dS values for 159 genes across the six species tree, binned by expression level

$$H = \begin{pmatrix} \alpha_b & 0 & 0 \\ 0 & \alpha_\beta & 0 \\ 0 & 0 & \alpha_\gamma \end{pmatrix}, \quad \Sigma = \begin{pmatrix} \sigma_b & 0 & 0 \\ 0 & \sigma_\beta & 0 \\ 0 & 0 & \sigma_\gamma \end{pmatrix} \tag{16}$$

If we assume that the root value is drawn from the stationary distribution, we have that

$$(V_{\text{phylog}})_{ij} = \frac{\sigma^2}{2\alpha}\left(1 - e^{-2\alpha t_{ij}}\right) \tag{17}$$

Each gene is assumed to have its own optimum value, given by θ_g, drawn from a normal distribution, $N(\bar{\theta}, \tau^2)$. The parameters of this normal distribution are optimized during fitting, and the hyper-prior is specified along with the priors for the other model parameters. We can integrate over the possible values of θ_g for each gene, to give a new variance-covariance matrix given by:

$$V_{ij} = (V_{\text{phylog}})_{ij} + V_\theta \tag{18}$$

bioRxiv preprint doi: https://doi.org/10.1101/2025.11.24.690267; this version posted November 24, 2025. The copyright holder for this preprint (which was not certified by peer review) is the author/funder, who has granted bioRxiv a license to display the preprint in perpetuity. It is made available under aCC-BY 4.0 International license.

Since initial fits gave high values for the diagonal elements of H (indicating low phylogenetic signal for many genes), we fitted a mixture model, following Chaix et al.[14]. For the 'outlier' distribution, we used a white-noise (wn) (normal) distribution, centered at $\bar{\theta}$, with variance $\sigma^2_{\mathrm{wn}} + \tau^2$, to represent a process of rapid mean-reversion with negligible phylogenetic signal. As in[14], we then optimized for a total likelihood given by:

$$\mathsf{LL} = p_{\mathrm{wn}}\mathsf{LL}_{\mathrm{out}} + (1 - p_{\mathrm{wn}})\mathsf{LL}_{\mathrm{in}}, \tag{19}$$

where $\mathsf{LL}_{\mathrm{in}}$ is the likelihood of the original model. We implemented this via an MCMC in rstan. The simulation results for MCMC fits to the selection strength, standard deviations and white noise probabilities are shown in Figure S6.

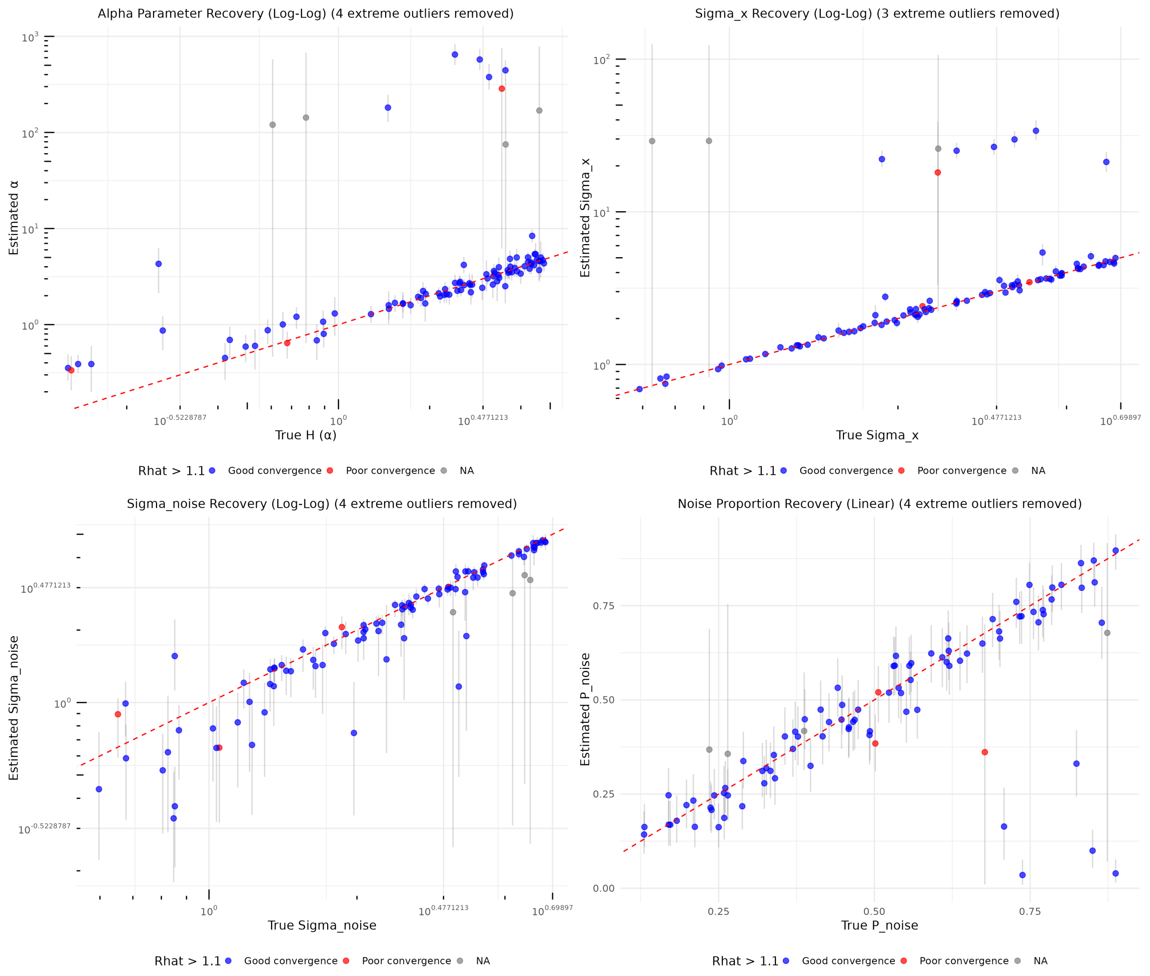

Figure S6: Estimated vs true parameters for simulated data under the independent model described in Section S3. The mean posterior values for the MCMC, are plotted against the known, simulated parameters. The red points showed poor convergence.

S3.1 Independent model results

We applied the model to the data described in Section S1. The posterior distributions for the most significant parameters are shown in Figure S7. The posteriors for all of the parameters are shown in Figure S8.

bioRxiv preprint doi: https://doi.org/10.1101/2025.11.24.690267; this version posted November 24, 2025. The copyright holder for this preprint (which was not certified by peer review) is the author/funder, who has granted bioRxiv a license to display the preprint in perpetuity. It is made available under aCC-BY 4.0 International license.

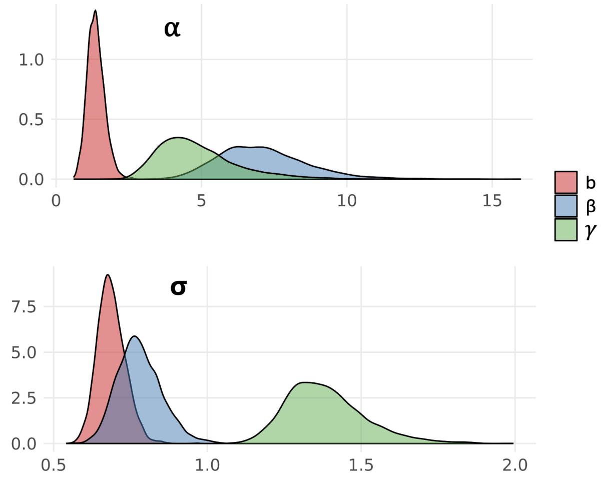

Figure S7: Posterior distributions for the selection strength (α) and the mutation strength (σ), for the independent evolution of the logarithms of the biophysical parameters: burst size (b, red), splicing rate (β, blue) and decay rate (γ, green).

S4 Three-parameter H models

We also investigated models of the above form, but with the off-diagonal H terms allowed to vary from the corresponding diagonal value. This is equivalent to allowing ϕ_b and ϕ_γ respectively to differ from one (see Section S2). This gives selection matrices:

$$H_b = \begin{pmatrix} \alpha_b & -\phi_b \alpha_b \\ 0 & \alpha_\gamma \end{pmatrix}, \qquad H_\gamma = \begin{pmatrix} \alpha_b & 0 \\ -\phi_\gamma \alpha_\gamma & \alpha_\gamma \end{pmatrix}, \tag{20}$$

and $\hat{X}$ values:

$$\hat{X}_b = \begin{pmatrix} \theta_b + \phi_b \theta_\gamma \\ \theta_\gamma \end{pmatrix}, \qquad \hat{X}_\gamma = \begin{pmatrix} \theta_b \\ \theta_\gamma + \phi_\gamma \theta_b \end{pmatrix}, \tag{21}$$

for the two models respectively. The other details of the models were identical to Section S2. Although these models had better AIC scores than those discussed in the main text, because of the required additional parameter, and the superior interpretability of the two-dimensional models, we have discussed those more at length.

S4.1 Simulations results: three-parameter models

As for the two-parameter H case, we simulate 100 datasets under each of hypotheses 1 and 2 (decay rate and burst size-driven), this time with the extra $\phi_{b,\gamma}$ parameters. The results for the three-parameter decay-rate-constrained model are shown in Figure S9, and the results for the three-parameter burst-size-constrained model are shown in Figure S10.

As before, we perform a simulated model comparison for the three-parameter H case. We show the distribution of AIC differences in favor of the true simulated model in Figure S11. We show the corresponding accuracy of differentiating between models using the AIC value in Figure S12.

bioRxiv preprint doi: https://doi.org/10.1101/2025.11.24.690267; this version posted November 24, 2025. The copyright holder for this preprint (which was not certified by peer review) is the author/funder, who has granted bioRxiv a license to display the preprint in perpetuity. It is made available under aCC-BY 4.0 International license.

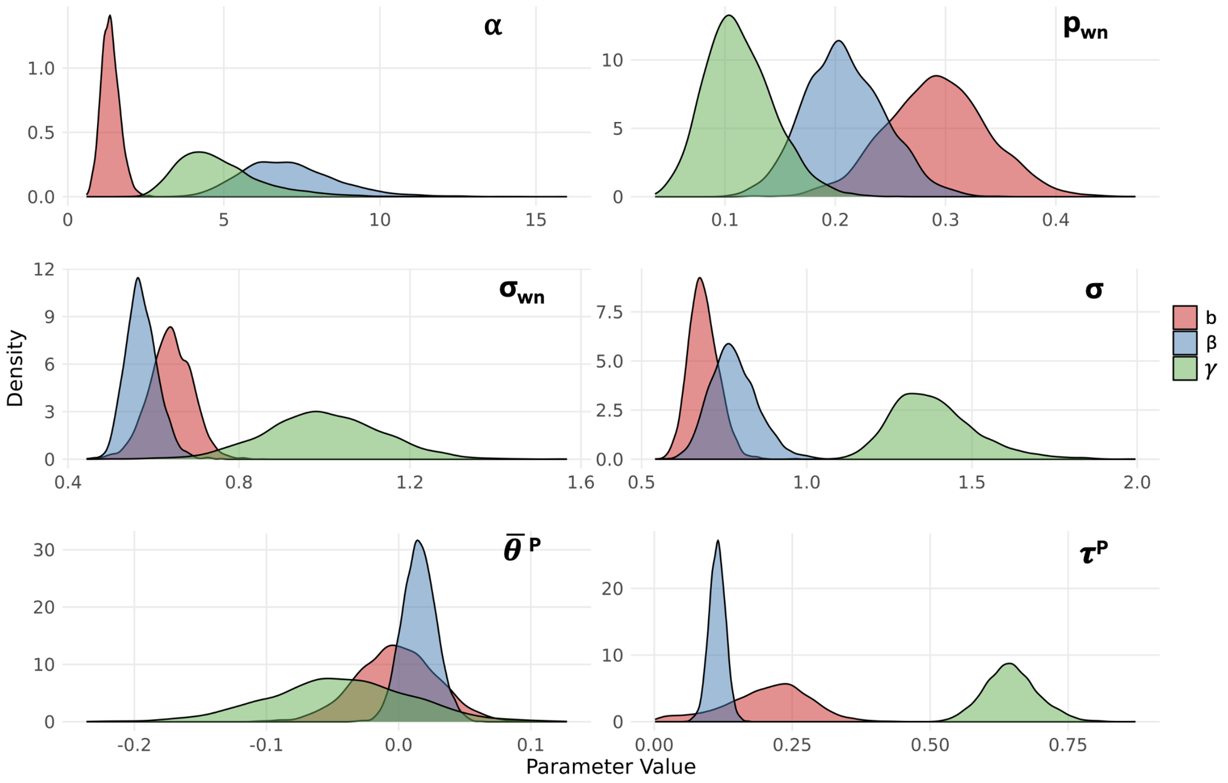

Figure S8: Posterior distributions for the selection strength (α) and mutation strength (σ) in the independent OU model. The mixture model probability, p_{wn}, the white-noise distribution standard deviation, σ_{wn}, and the parameters of the assumed underlying distribution for the gene optima and white-noise means ($\bar{\theta}^P, \tau^P$) are also shown. These are the parameters governing the independent evolution of the logarithms of the biophysical parameters: burst size (b, red), splicing rate (β, blue) and decay rate (γ, green).

S4.2 Data fits: three-parameter models

We include the fitted parameter values for the three-parameter H models in Table 2. Note that the AIC is again much better for the decay-rate-constrained model, and p_{wn} goes to the upper bound in the burst-size-constrained model.

Table 2: Three-parameter model fitted parameters

Model	α_b	α_γ	$\phi_{b,\gamma}$	σ_b	σ_γ	p_{wn}	AIC
γ-constrained	130.2	4.21	0.717	2.20	1.33	0.226	1972
b-constrained	1.91	4.68	1.84	0.567	0.00676	0.9	3166

S5 Theta integration

For a vector of traits (e.g. (b, β, γ)), we consider the distribution of values for a single species given a constant optimum, θ. We define:

$$\boldsymbol{f}(\boldsymbol{x}_t, t) \equiv e^{\hat{H}t}\boldsymbol{x}_t, \tag{22}$$

bioRxiv preprint doi: https://doi.org/10.1101/2025.11.24.690267; this version posted November 24, 2025. The copyright holder for this preprint (which was not certified by peer review) is the author/funder, who has granted bioRxiv a license to display the preprint in perpetuity. It is made available under aCC-BY 4.0 International license.

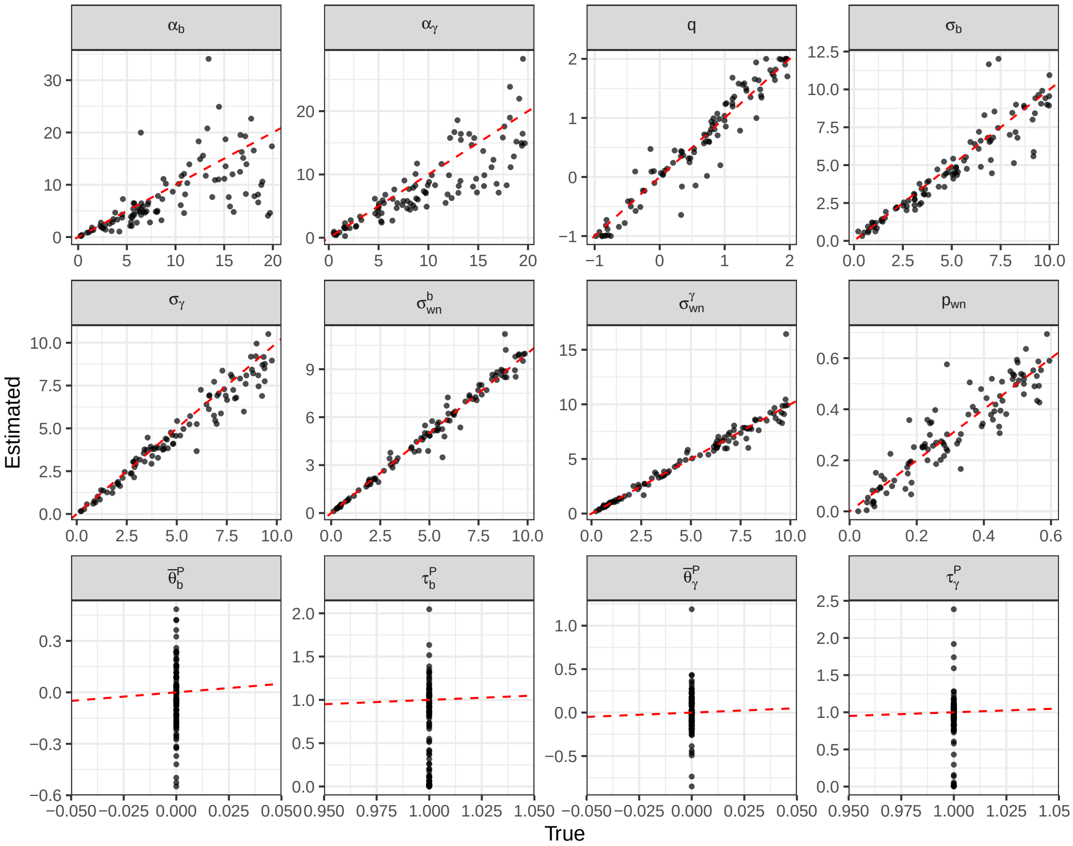

Figure S9: Fitted vs true parameters for the decay-rate-constrained, three-parameter H model.

185 where $\hat{H}$ is the selection matrix. As in the one-trait case, we have:

$$d\boldsymbol{f} = e^{\hat{H}t} d\boldsymbol{x}_t + \hat{H} e^{\hat{H}t} \boldsymbol{x}_t. \tag{23}$$

186 Then, using the OU equation

$$d\boldsymbol{x}_t = -\hat{H}(\boldsymbol{x}_t - \boldsymbol{\theta})dt + \hat{\Sigma}\boldsymbol{dW}_t, \tag{24}$$

187 where $\boldsymbol{dW}$ is a vector whose components are independent instantiations of the random vari-
188 able, dW (Wiener process), and correlations between changes in the different variables of $\boldsymbol{x}_t$ can
189 be introduced via off-diagonal elements in Σ. This gives:

$$\begin{aligned} d\boldsymbol{f} &= e^{\hat{H}t}\left(-\hat{H}(\boldsymbol{x}_t - \boldsymbol{\theta})dt + \hat{\Sigma}\boldsymbol{dW}_t\right) + \hat{H}e^{\hat{H}t}\boldsymbol{x}_t \\ &= e^{\hat{H}t}\hat{H}\boldsymbol{\theta}dt + e^{\hat{H}t}\hat{\Sigma}\boldsymbol{dW}_t, \end{aligned} \tag{25}$$

190 where, for the second equality, we have used the commutation of $e^{\hat{H}t}$ and $\hat{H}$. Using the
191 convenient properties of the matrix exponential, we can solve this via:

bioRxiv preprint doi: https://doi.org/10.1101/2025.11.24.690267; this version posted November 24, 2025. The copyright holder for this preprint (which was not certified by peer review) is the author/funder, who has granted bioRxiv a license to display the preprint in perpetuity. It is made available under aCC-BY 4.0 International license.

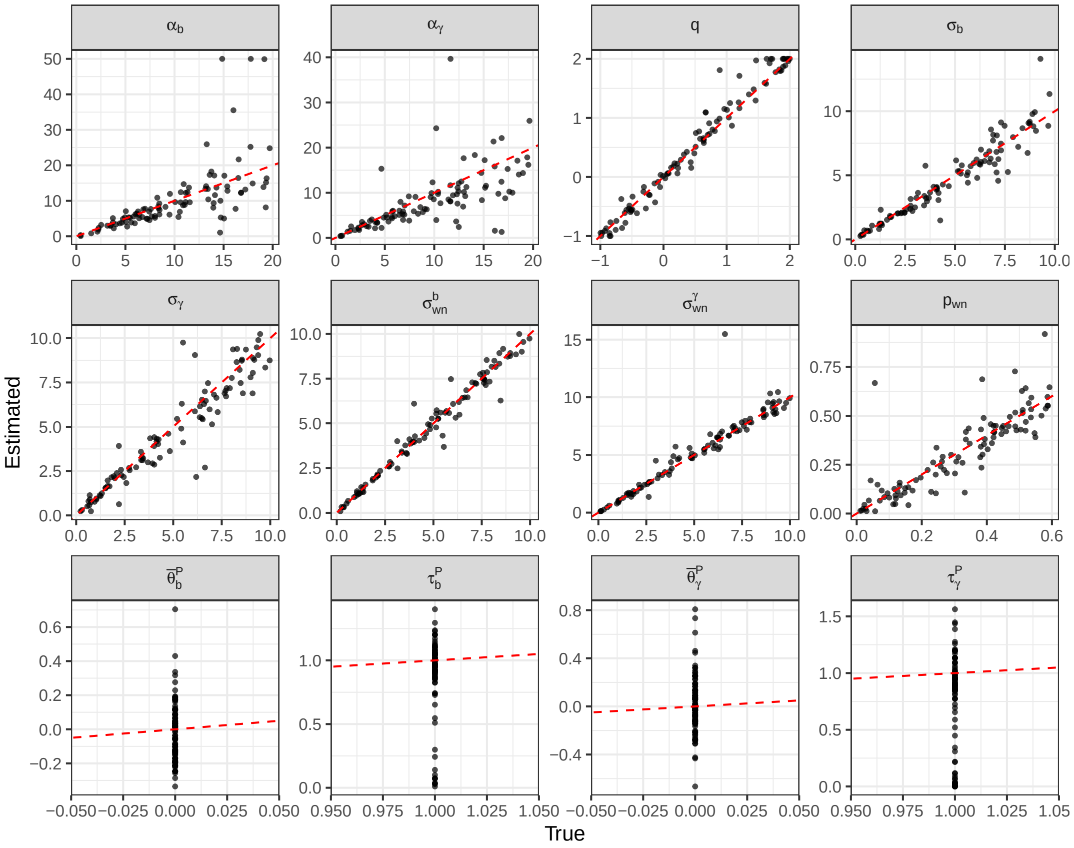

Figure S10: Fitted vs true parameters for the burst-size-constrained, three-parameter H model.

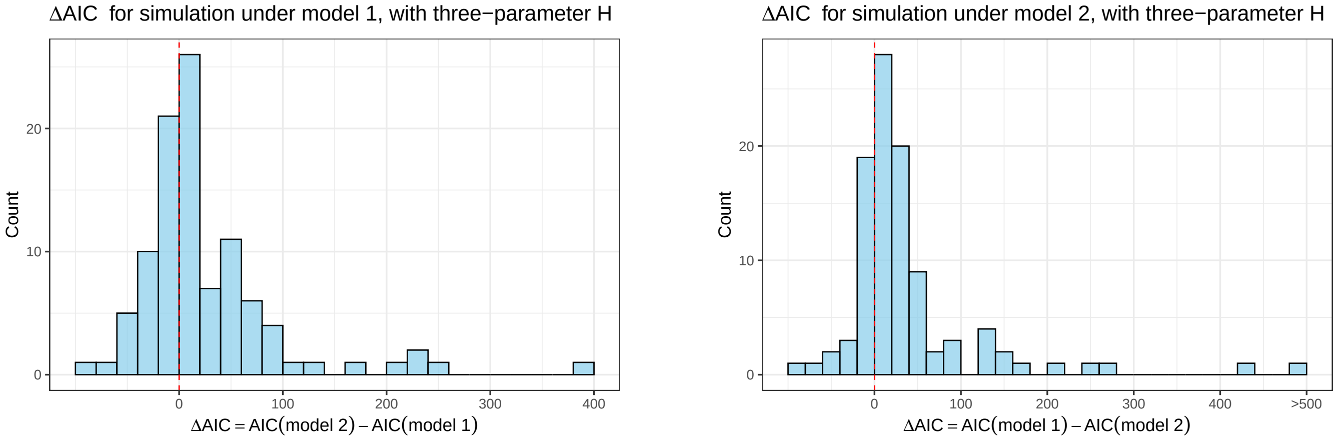

Figure S11: Distributions of AIC differences in favor of the simulated model, for datasets simulated under the three-parameter H versions of the decay-rate-constrained model **(left)** and the burst-size-constrained model **(right)**, fitted under both models.

$$\begin{aligned}
\boldsymbol{f} &= \boldsymbol{f}(0) + \int_0^t d\boldsymbol{f} \\
&= \boldsymbol{x_0} + \left[\hat{H}\hat{H}^{-1}e^{\hat{H}s}\boldsymbol{\theta}\right]_0^t + \int_0^t e^{\hat{H}s}\hat{\Sigma}\boldsymbol{dW}_s \\
&= \boldsymbol{x_0} + \left(e^{\hat{H}t} - \hat{I}\right)\boldsymbol{\theta} + \int_0^t e^{\hat{H}s}\hat{\Sigma}\boldsymbol{dW}_s, \qquad (26)
\end{aligned}$$

bioRxiv preprint doi: https://doi.org/10.1101/2025.11.24.690267; this version posted November 24, 2025. The copyright holder for this preprint (which was not certified by peer review) is the author/funder, who has granted bioRxiv a license to display the preprint in perpetuity. It is made available under aCC-BY 4.0 International license.

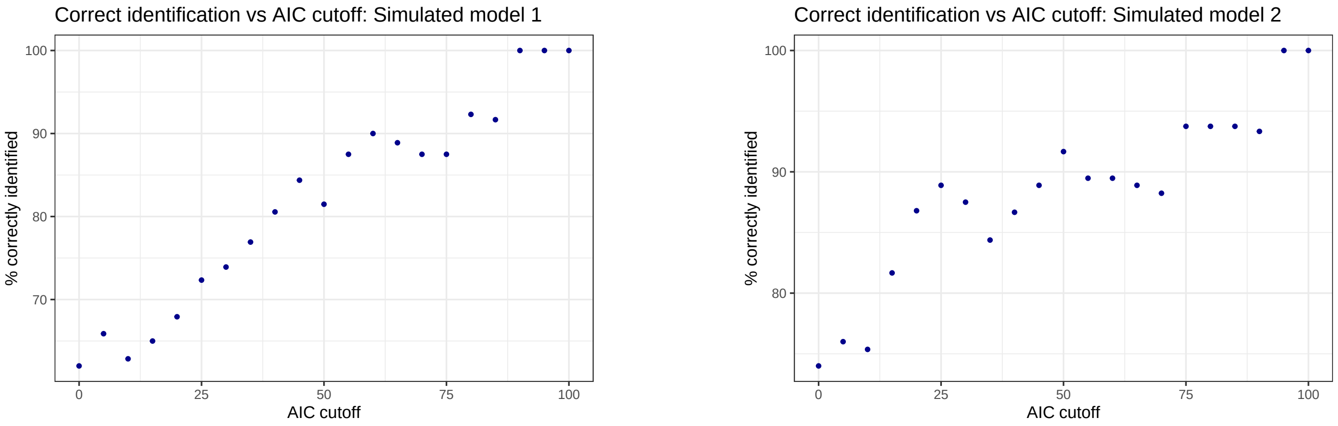

Figure S12: Accuracy of model identifications made at various AIC cutoffs, for datasets simulated under the three-parameter H versions of the decay-rate-constrained model **(left)** and the burst-size-constrained model **(right)**, fitted under both models. The accuracy is defined as the fraction of model fits with AIC differences above each cutoff which would be attributed to the correct model.

for s our dummy time variable. Then, by the definition of $\boldsymbol{f}$, (and the commutation of $\hat{H}$ with itself and its inverse), we have:

$$\begin{aligned} \boldsymbol{x}_t &= e^{-\hat{H}t}\boldsymbol{f}(\boldsymbol{x}_t, t) \\ \implies \boldsymbol{x}_t &= e^{-\hat{H}t}\boldsymbol{x_0} + \left(\hat{I} - e^{-\hat{H}t}\right)\boldsymbol{\theta} + \int_0^t e^{-\hat{H}(t-s)}\hat{\Sigma}\boldsymbol{dW}_s. \end{aligned} \tag{27}$$

Note that in the limit $t \to \infty$, for positive definite $\hat{H}$, the first term vanishes, and we are left with a linear sum of the independent normal random variables, dW_s, which make up $\boldsymbol{dW}_s$. Since each component of $\boldsymbol{x}_t$ is a linear comination of the dW values, the vector $\boldsymbol{x}_t$ has a multivariate normal distribution. We can proceed to calculate the moments of this distribution. We find, for a single tip, i, at time $t = t_i$,

$$\mathbb{E}(\boldsymbol{x}_{t_i}) = e^{-\hat{H}t_i}\mathbb{E}(\boldsymbol{x_0}) + \left(\hat{I} - e^{-\hat{H}t_i}\right)\boldsymbol{\theta}, \tag{28}$$

since $\mathbb{E}(\boldsymbol{dW_t}) = \boldsymbol{0}$ for all t, and $\boldsymbol{\theta}$ and $\hat{H}$ are fixed parameters of the system. We now consider the variance-covariance of the different traits at a single tip. We use Greek indices to indicate the different traits at a tip, (and Latin indices to indicate the tip (extant species)). Since $\boldsymbol{x_0}$ and the stochastic path integral are independent random variables (and the middle term is constant), we have:

$$\text{Var}(\boldsymbol{x}_{t_i}) = \text{Var}\left(e^{-\hat{H}t_i}\boldsymbol{x_0}\right) + \text{Var}\left(\int_0^{t_i} e^{-\hat{H}(t_i-s)}\hat{\Sigma}\boldsymbol{dW}_s\right). \tag{29}$$

First we focus on the $\boldsymbol{x_0}$ term. By simple properties of matrices we have:

$$\text{Var}\left(e^{-\hat{H}t_i}\boldsymbol{x_0}\right) = e^{-\hat{H}t_i}\hat{V}_{x_0}\left(e^{-\hat{H}t_i}\right)^T, \tag{30}$$

bioRxiv preprint doi: https://doi.org/10.1101/2025.11.24.690267; this version posted November 24, 2025. The copyright holder for this preprint (which was not certified by peer review) is the author/funder, who has granted bioRxiv a license to display the preprint in perpetuity. It is made available under aCC-BY 4.0 International license.

for $\hat{V}_{x_0}$ the variance-covariance matrix for $\boldsymbol{x_0}$. Then we focus on the integral term. As above, the expectations of all components of $\boldsymbol{dW}$ are zero. For generic traits α and β we therefore have:

$$\text{Cov}_i^{\text{integral}}(\alpha,\beta) = \mathbb{E}\left[\left(\int_0^{t_i} e^{-\hat{H}(t_i-s)}\hat{\Sigma}\boldsymbol{dW}_s\right)_\alpha \left(\int_0^{t_i} e^{-\hat{H}(t_i-u)}\hat{\Sigma}\boldsymbol{dW}_u\right)_\beta\right]. \tag{31}$$

Since each individual dW_s is assumed independent from the last, and has zero expectation, we have:

$$\begin{aligned}
\text{Cov}_i^{\text{integral}}(\alpha,\beta) &= \mathbb{E}\left[\int_0^{t_i} \left(e^{-\hat{H}(t_i-s)}\Sigma\boldsymbol{dW}_s\right)_\alpha \left(e^{-\hat{H}(t_i-s)}\Sigma\boldsymbol{dW}_s\right)_\beta\right] \\
&= \mathbb{E}\left[\int_0^{t_i} \sum_{\gamma\nu\epsilon\mu} e^{-\hat{H}(t_i-s)}{}_{\alpha\gamma}\hat{\Sigma}_{\gamma\nu}(\boldsymbol{dW}_s)_\nu e^{-\hat{H}(t_i-s)}{}_{\beta\epsilon}\hat{\Sigma}_{\epsilon\mu}(\boldsymbol{dW}_s)_\mu\right] \\
&= \int_0^{t_i} \sum_{\gamma\nu\epsilon\mu} e^{-\hat{H}(t_i-s)}{}_{\alpha\gamma}\hat{\Sigma}_{\gamma\nu} e^{-\hat{H}(t_i-s)}{}_{\beta\epsilon}\hat{\Sigma}_{\epsilon\mu}\delta_{\mu\nu} ds \\
&= \int_0^{t_i} \sum_{\gamma\epsilon\mu} e^{-\hat{H}(t_i-s)}{}_{\alpha\gamma}\hat{\Sigma}_{\gamma\mu} e^{-\hat{H}(t_i-s)}{}_{\beta\epsilon}\hat{\Sigma}_{\epsilon\mu} ds \\
&= \sum_{\gamma\epsilon\mu} \hat{\Sigma}_{\gamma\mu}\hat{\Sigma}_{\epsilon\mu}\int_0^{t_i} e^{-\hat{H}(t_i-s)}{}_{\alpha\gamma} e^{-\hat{H}(t_i-s)}{}_{\beta\epsilon} ds \\
&= \int_0^{t_i} \left[e^{-\hat{H}(t_i-s)}\hat{\Sigma}\hat{\Sigma}^T (e^{-\hat{H}(t_i-s)})^T\right]_{\alpha\beta} ds
\end{aligned} \tag{32}$$

For notes on solving this, see Jonathan Goodman, NYU[15]. Next, we consider the covariance between traits α and β, between two *different* species i and j. We have:

$$\text{Cov}_{ij\alpha\beta} = \text{Cov}_{ij}^{\text{integral}}(\alpha,\beta) + \left[e^{-\hat{H}t_i}\hat{V}_{x_0}\left(e^{-\hat{H}t_j}\right)^T\right]_{\alpha\beta}. \tag{33}$$

Recall that the stationary variance satisfies:

$$\hat{V}_{\text{stat}}\hat{H}^T + \hat{H}\hat{V}_{\text{stat}} = \hat{\Sigma}\hat{\Sigma}^T. \tag{34}$$

We have already shown that the stationary distribution is multivariate Gaussian. If $\boldsymbol{x_0}$ is drawn from this distribution, it will therefore be distributed via:

$$\boldsymbol{x_0} \sim N(\boldsymbol{\theta}, \hat{V}_{\text{stat}}), \tag{35}$$

and the entire process is also multivariate normal given by:

$$\boldsymbol{x}_t \sim N(\boldsymbol{\theta}, \hat{V}), \tag{36}$$

using again that $\hat{H}^{-1}$ and $e^{-\hat{H}t}$ commute to calculate the mean, and $\hat{V}$ is the full covariance matrix. If $\boldsymbol{\theta}$ is another random variable chosen independently from a prior, and the previous distribution of $\boldsymbol{x}_t$ is in fact $\boldsymbol{x}_t|\boldsymbol{\theta}$, we consider again:

bioRxiv preprint doi: https://doi.org/10.1101/2025.11.24.690267; this version posted November 24, 2025. The copyright holder for this preprint (which was not certified by peer review) is the author/funder, who has granted bioRxiv a license to display the preprint in perpetuity. It is made available under aCC-BY 4.0 International license.

$$\boldsymbol{x}_t = e^{-\hat{H}t}\boldsymbol{x_0} + \left(\hat{I} - e^{-\hat{H}t}\right)\boldsymbol{\theta} + \int_0^t e^{-\hat{H}(t-s)}\hat{\Sigma}\boldsymbol{dW}_s \tag{37}$$

Since the last term is linear combinations of the same dW, it is clearly multivariate normal, and independent from the other two terms. $\boldsymbol{\theta}$ is drawn from a normal prior, $N(\mu_\theta, \hat{V}_\theta)$. This determines the distribution of the r.v. $\boldsymbol{x_0}$. This distribution is determined by imagining the stationary version of the equation, where $t \to \infty$:

$$\boldsymbol{x}_{\text{stat}} = \boldsymbol{\theta} + \lim_{t\to\infty}\int_0^t e^{-\hat{H}(t-s)}\hat{\Sigma}\boldsymbol{dW}_s. \tag{38}$$

This stationary distribution clearly would be multivariate normal with expectation $\boldsymbol{\theta}$ and some stationary variance $\hat{V}_{\text{stat}}$, which depends on the model parameters. To show that $\boldsymbol{x_0}$ is multivariate normal with $\boldsymbol{\theta}$, consider the sum of the two variables. Consider $\boldsymbol{x_0} = \boldsymbol{\theta} + \epsilon$, with $\epsilon \sim N(0, \hat{V}_{\text{stat}})$. For deterministic α, β and *independent* $\boldsymbol{\theta}$ and ϵ, we have $\alpha\boldsymbol{x_0} + \beta\boldsymbol{\theta} = (\alpha+\beta)\boldsymbol{\theta} + \alpha\boldsymbol{\epsilon}$. Clearly, the whole of $\boldsymbol{x}_t$ is still a multivariate normal random variable. The expected value is given by:

$$\begin{aligned}\mathbb{E}(\boldsymbol{x}_t) &= e^{-\hat{H}t}\mathbb{E}(\boldsymbol{x_0}) + \left(\hat{I} - e^{-\hat{H}t}\right)\boldsymbol{\theta}\\ &= e^{-\hat{H}t}\mu_\theta + \left(\hat{I} - e^{-\hat{H}t}\right)\mu_\theta\\ &= \mu_\theta,\end{aligned} \tag{39}$$

where we have used that $\mathbb{E}(\boldsymbol{dW}) = \mathbf{0}$. Considering $\boldsymbol{\theta}$ as a random variable changes the variance-covariance matrix of the distribution, since now we have to consider:

$$\text{Var}_i\left(e^{-\hat{H}t_i}\boldsymbol{x_0} + \left(\hat{I} - e^{-\hat{H}t_i}\right)\boldsymbol{\theta}\right), \tag{40}$$

retaining the separation of this term from the integral term, since the two are independent. Considering the decomposition above, we get:

$$\text{Var}_i\left(e^{-\hat{H}t}(\boldsymbol{\theta}+\boldsymbol{\epsilon}) + \left(\hat{I} - e^{-\hat{H}t}\right)\boldsymbol{\theta}\right) = \text{Var}_i\left(e^{-\hat{H}t}\boldsymbol{\epsilon} + \boldsymbol{\theta}\right). \tag{41}$$

Considering this, trait-component-wise, we have:

$$\begin{aligned}\left[\text{Var}_i\left(e^{-\hat{H}t}\boldsymbol{\epsilon}+\boldsymbol{\theta}\right)\right]_{\alpha\beta} &= \left\langle \left(e^{-\hat{H}t}\boldsymbol{\epsilon}+\boldsymbol{\theta}\right)_\alpha \left(e^{-\hat{H}t}\boldsymbol{\epsilon}+\boldsymbol{\theta}\right)_\beta \right\rangle - \left\langle \left(e^{-\hat{H}t}\boldsymbol{\epsilon}+\boldsymbol{\theta}\right)_\alpha \right\rangle \left\langle \left(e^{-\hat{H}t}\boldsymbol{\epsilon}+\boldsymbol{\theta}\right)_\beta \right\rangle\\ &= \left\langle \left(\sum_\gamma (e^{-\hat{H}t})_{\alpha\gamma}\boldsymbol{\epsilon}_\gamma + \boldsymbol{\theta}_\alpha\right)\left(\sum_\mu (e^{-\hat{H}t})_{\beta\mu}\boldsymbol{\epsilon}_\mu + \boldsymbol{\theta}_\beta\right)\right\rangle - \mu_\theta^2\mathbf{1}\\ &= \left\langle \sum_\gamma (e^{-\hat{H}t})_{\alpha\gamma}\boldsymbol{\epsilon}_\gamma \sum_\mu (e^{-\hat{H}t})_{\beta\mu}\boldsymbol{\epsilon}_\mu + \boldsymbol{\theta}_\alpha\boldsymbol{\theta}_\beta\right\rangle - \mu_\theta^2\mathbf{1}\\ &= \sum_{\gamma\mu}(e^{-\hat{H}t})_{\alpha\gamma}(e^{-\hat{H}t})_{\beta\mu}\langle\boldsymbol{\epsilon}_\gamma\boldsymbol{\epsilon}_\mu\rangle + \hat{V}_{\boldsymbol{\theta}}\\ &= (e^{-\hat{H}t_i})\hat{V}_{\text{stat}}(e^{-\hat{H}t_i})^T + \hat{V}_{\boldsymbol{\theta}}\end{aligned} \tag{42}$$

bioRxiv preprint doi: https://doi.org/10.1101/2025.11.24.690267; this version posted November 24, 2025. The copyright holder for this preprint (which was not certified by peer review) is the author/funder, who has granted bioRxiv a license to display the preprint in perpetuity. It is made available under aCC-BY 4.0 International license.

Since the integral term is unchanged, this simply represents summing the original variance-covariance matrix with the variance-covariance matrix for $\boldsymbol{\theta}$. Since the $\boldsymbol{x_0}$ and $\boldsymbol{\theta}$ values are common between species, the components of $\text{Cov}_{ij\alpha\beta}$, between different species i and j, will also pick up these same covariance terms. This will give the full, overall formula:

$$\text{Cov}_{ij\alpha\beta} = \text{Cov}_{ij}^{\text{integral}}(\alpha, \beta) + \left[e^{-\hat{H}t_i} \hat{V}_{x_0} \left(e^{-\hat{H}t_j} \right)^T \right]_{\alpha\beta} + \left[\hat{V}_{\boldsymbol{\theta}} \right]_{\alpha\beta}. \tag{43}$$

This allows us to effectively integrate over a Gaussian prior on the optima, by using the prior distribution covariance in $\hat{V}_\theta$, and considering a new Gaussian distribution for $\boldsymbol{x_t}$ with a covariance given by Eq. 43.

bioRxiv preprint doi: https://doi.org/10.1101/2025.11.24.690267; this version posted November 24, 2025. The copyright holder for this preprint (which was not certified by peer review) is the author/funder, who has granted bioRxiv a license to display the preprint in perpetuity. It is made available under aCC-BY 4.0 International license.

References

1. Jiao, A., Zhang, C., Wang, X., Sun, L., Liu, H., Su, Y., Lei, L., Li, W., Ding, R., Ding, C., Dou, M., Tian, P., Sun, C., Yang, X., Zhang, L., and Zhang, B. (2024). Single-cell sequencing reveals the evolution of immune molecules across multiple vertebrate species. Journal of Advanced Research *55*, 73–87. URL: `https://doi.org/10.1016/j.jare.2023.02.017`. doi: `10.1016/j.jare.2023.02.017`. Epub 2023 Mar 4.

2. Sullivan, D.K. et al. (2025). kallisto, bustools and kb-python for quantifying bulk, single-cell and single-nucleus rna-seq. Nature Protocols *20*, 587–607. doi: `10.1038/s41596-024-01057-0`.

3. Kinsella, R.J., Kähäri, A., Haider, S., Zamora, J., Proctor, G., Spudich, G., Almeida-King, J., Staines, D., Derwent, P., Kerhornou, A., Kersey, P., and Flicek, P. (2011). Ensembl BioMarts: A hub for data retrieval across taxonomic space. Database: The Journal of Biological Databases and Curation *2011*, bar030. doi: `10.1093/database/bar030`.

4. Gorin, G., and Pachter, L. (2023). Distinguishing biophysical stochasticity from technical noise in single-cell RNA sequencing using Monod. bioRxiv. URL: `https://www.biorxiv.org/content/early/2023/04/17/2022.06.11.495771`. doi: `10.1101/2022.06.11.495771`.

5. Kumar, S., Suleski, M., Craig, J.M., Kasprowicz, A.E., Sanderford, M., Li, M., Stecher, G., and Hedges, S.B. (2022). TimeTree 5: An Expanded Resource for Species Divergence Times. Molecular Biology and Evolution *39*, msac174. doi: `10.1093/molbev/msac174`.

6. Cope, A.L., Schraiber, J.G., and Pennell, M. (2025). Macroevolutionary divergence of gene expression driven by selection on protein abundance. Science *387*. doi: `10.1126/science.ads2658`.

7. Nash, J.C., and Varadhan, R. (2011). Unifying optimization algorithms to aid software system users: Optimx for R. Journal of Statistical Software *43*, 1–14. doi: `10.18637/jss.v043.i09`.

8. Mitov, V., Bartoszek, K., Asimomitis, G., and Stadler, T. (2020). Fast likelihood calculation for multivariate gaussian phylogenetic models with shifts. Theoretical Population Biology *131*, 66–78. URL: `https://doi.org/10.1016/j.tpb.2019.11.005`. doi: `10.1016/j.tpb.2019.11.005`.

9. Dyer, S.C., Austine-Orimoloye, O., Azov, A.G., Barba, M., Barnes, I., Barrera-Enriquez, V.P., Becker, A., Bennett, R., Beracochea, M., Berry, A., Bhai, J., Bhurji, S.K., Boddu, S., Branco Lins, P.R., Brooks, L., Ramaraju, S.B., Campbell, L.I., Martinez, M.C., Charkhchi, M., Cortes, L.A., Davidson, C., Denni, S., Dodiya, K., Donaldson, S., El Houdaigui, B., El Naboulsi, T., Falola, O., Fatima, R., Genez, T., Martinez, J.G., Gurbich, T., Hardy, M., Hollis, Z., Hunt, T., Kay, M., Kaykala, V., Lemos, D., Lodha, D., Mathlouthi, N., Merino, G.A., Merritt, R., Mirabueno, L.P., Mushtaq, A., Hossain, S.N., Pérez-Silva, J.G., Perry, M., Piližota, I., Poppleton, D., Prosovetskaia, I., Raj, S., Salam, A.I.A., Saraf, S., Saraiva-Agostinho, N., Sinha, S., Sipos, B., Sitnik, V., Steed, E., Suner, M.M., Surapaneni, L., Sutinen, K., Tricomi, F.F., Tsang, I., Urbina-Gómez, D., Veidenberg, A., Walsh, T.A., Willhoft, N.L., Allen, J., Alvarez-Jarreta, J., Chakiachvili, M., Cheema, J., da Rocha, J.B., De Silva, N.H., Giorgetti, S., Haggerty, L., Ilsley, G.R., Keatley, J., Loveland, J.E., Moore, B., Mudge, J.M., Naamati, G., Tate, J., Trevanion, S.J., Winterbottom, A., Flint, B., Frankish, A., Hunt, S.E., Finn, R.D., Freeberg, M.A., Harrison, P.W., Martin, F.J., and Yates, A.D. (2024). Ensembl 2025. Nucleic Acids Research *53*, D948–D957. doi: `10.1093/nar/gkae1071`.

bioRxiv preprint doi: https://doi.org/10.1101/2025.11.24.690267; this version posted November 24, 2025. The copyright holder for this preprint (which was not certified by peer review) is the author/funder, who has granted bioRxiv a license to display the preprint in perpetuity. It is made available under aCC-BY 4.0 International license.

10. Katoh, K., and Standley, D.M. (2013). MAFFT Multiple Sequence Alignment Software Version 7: Improvements in Performance and Usability. Molecular Biology and Evolution *30*, 772–780.

11. Suyama, M., Torrents, D., and Bork, P. (2006). PAL2NAL: robust conversion of protein sequence alignments into the corresponding codon alignments. Nucleic Acids Research *34*, W609–W612.

12. Yang, Z. (2007). PAML 4: Phylogenetic Analysis by Maximum Likelihood. Molecular Biology and Evolution *24*, 1586–1591.

13. Álvarez Carretero, S., Kapli, P., and Yang, Z. (2023). Beginner's Guide on the Use of PAML to Detect Positive Selection. Molecular Biology and Evolution *40*, msad041.

14. Chaix, R., Somel, M., Kreil, D.P., Khaitovich, P., and Lunter, G.A. (2008). Evolution of primate gene expression: Drift and corrective sweeps? Genetics *180*, 1379–1389. URL: https://doi.org/10.1534/genetics.108.089623. doi: 10.1534/genetics.108.089623.

15. (). . URL: https://math.nyu.edu/~goodman/teaching/MonteCarlo07/notes/sde2.pdf.
